# Trigeminal Neuralgia and Hemifacial Spasms Due to Vertebrobasilar Dolichoectasia: A Case Report

**DOI:** 10.7759/cureus.32148

**Published:** 2022-12-03

**Authors:** Rakan F Alotaibi, Ali H Al Mudhi, Abdulrahman M Almousa, Mohammed A Albaqshi, Mustafa Alturki

**Affiliations:** 1 General Practice, Imam Abdulrahman Bin Faisal University, Dammam, SAU; 2 General Practice, Wroclaw Medical University, Wroclaw, POL; 3 Surgery, Al-Nairyah General Hospital, Nairyah, SAU

**Keywords:** brain mri, mri imaging, case report, magnetic resonance imaging, facial spasm, vertebrobasilar dolichoectasia, trigeminal neuralgia

## Abstract

Trigeminal neuralgia is a rare condition characterized by brief, recurrent episodes of severe, unilateral, sharp pain limited to the sensory distribution of the trigeminal nerve. Neurovascular compression in the cisternal segment of the trigeminal nerve is considered the most common cause. Here, we present the case of an elderly man who had a two-year history of electric shock-like pain involving the right side of the face and associated facial spasms. The patient had a long-standing history of hypertension, diabetes mellitus, dyslipidemia, and previous coronary artery bypass graft surgery. The patient underwent magnetic resonance imaging, which revealed abnormal dilatation and tortuosity of the vertebral and basilar arteries, which resulted in compression of the facial and trigeminal nerves along with brainstem compression. Such findings were consistent with the diagnosis of vertebrobasilar dolichoectasia. The patient was given medical treatment in the form of carbamazepine, which resulted in satisfactory improvement in his symptoms. Vertebrobasilar dolichoectasia is a rare cause of neurovascular compression of the trigeminal and facial nerves that can lead to trigeminal neuralgia and facial hemispasm. Medical management should be attempted first, particularly in those patients who are not candidates for surgical interventions.

## Introduction

Trigeminal neuralgia is a clinical condition characterized by brief, recurrent episodes of intense, unilateral shock-like pain with sudden onset and termination that are limited to the sensory distribution of the trigeminal nerve [1]. While many pathologies along the anatomic segments of the trigeminal nerve can cause trigeminal neuralgia, neurovascular compression at the cisternal segment of the trigeminal nerve is considered the most common cause. Other causes include brainstem lesions that may be infarctions, demyelinations, or neoplastic. The root entry zone, which is the transition zone between the central and peripheral myelination zones, is considered the most vulnerable zone to pressure. It is thought that neurovascular compression leads to a focal area of demyelination, which generates ectopic neural impulses and leads to the perception of painful episodes by light touches on the facial zones [2]. Neurovascular compression is typically caused by the superior cerebellar artery or the anterior inferior cerebellar artery. Other rare causes of neurovascular compression reported include aneurysms, arteriovenous malformations, vertebrobasilar dolichoectasia, arteriovenous fistulas, and compression by veins [3]. In such conditions, other cranial nerves, such as the facial nerve, may also get compressed, resulting in additional symptoms like facial spasms [3-4]. Here, we report a case of trigeminal neuralgia caused by vertebrobasilar dolichoectasia in an elderly man.

## Case presentation

We present the case of a 77-year-old man who came to our emergency department with severe episodes of right-sided headaches. The headache was described as a sharp, electric shock-like pain that lasted for 30 seconds and occurred multiple times a day. There were no preceding warning symptoms before the headache episodes. The patient reported that the episodes were precipitated by a light touch on the face. He reported that he had been experiencing this headache for two years, but recently, the headache episodes became more severe and occurred more frequently. The headache was not relieved with nonsteroidal anti-inflammatory drugs. The associated symptoms included involuntary contractions of the facial muscles on the right side and mild nausea. The patient reported that he was completely pain-free between episodes. He scored his pain at nine out of 10 on the severity scale.

His past medical history was remarkable for hypertension, diabetes mellitus, and dyslipidemia for more than 30 years. A review of the medical records revealed that these conditions were poorly controlled. There was no history of transient ischemic attacks or seizures. The patient had undergone coronary artery bypass graft surgery 10 years ago. The patient had been a heavy smoker, smoking at least two packs a day for more than 40 years. His medications included aspirin 75 mg, metformin 1000 mg, bisoprolol 5 mg, perindopril 5 mg, and atorvastatin 20 mg. The family history was unremarkable. The patient was a retired school teacher and lived independently with his wife.

On examination, the patient appeared to be in pain. The vital signs included a normal temperature of 36.8°C, a pulse rate of 90 beats per minute, a respiratory rate of 14 breaths per minute, a blood pressure of 150/100 mmHg, and a normal oxygen saturation of 98% on room air. The patient was alert, conscious, and fully oriented. A neurological examination revealed no focal neurological deficit. Examination of the upper and lower extremities revealed normal tone, power, and reflexes. A cranial nerve examination revealed normal findings apart from mildly increased touch sensitivity on the right side of the face. The clinical features were suggestive of trigeminal neuralgia.

**Figure 1:**
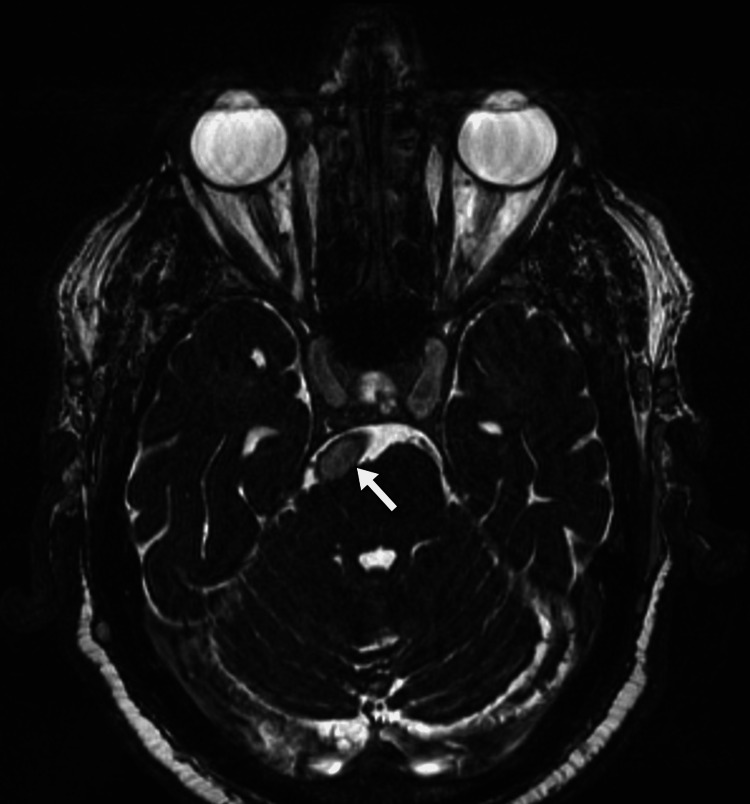
An axial image of the patient's brain MRI in the constructive interference of the CISS sequence shows abnormal dilatation of the basilar artery (arrow) with a compressive effect on the pons. CISS: constructive interference in steady state; MRI: magnetic resonance imaging.

**Figure 2:**
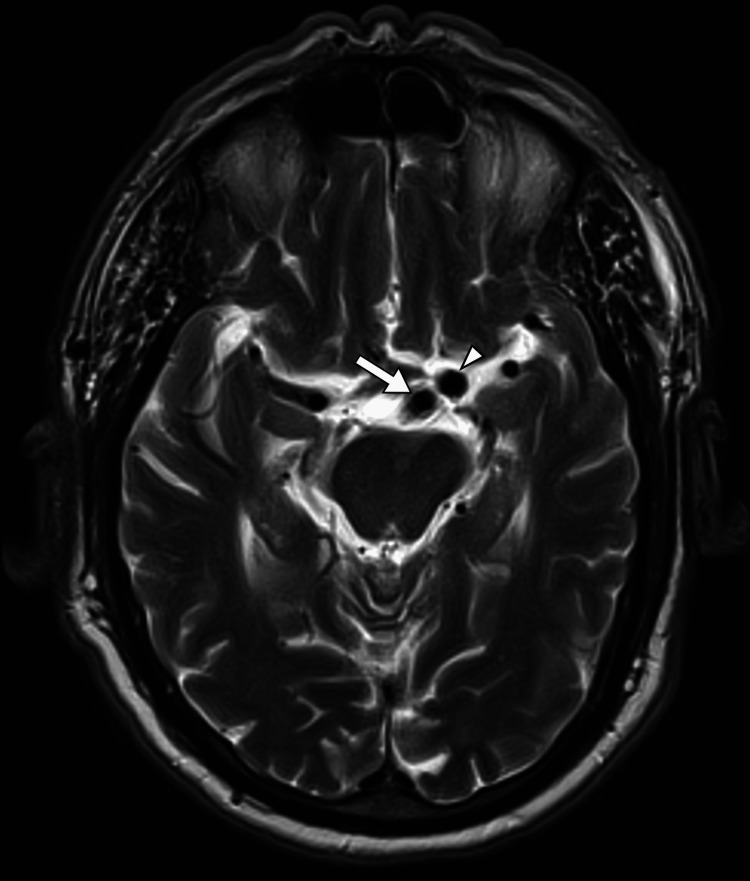
An axial T2-weighted image of the patient's brain MRI shows abnormal dilatation of the basilar artery (arrow), with its tip in the suprasellar cistern adjacent to the left middle cerebral artery (arrowhead).

Given the long-standing history of headaches and the presence of localizing signs on physical examination, the patient was referred to undergo a brain MRI to exclude any space-occupying lesion. The scan revealed that the vertebral and basilar arteries were elongated, dilated, and tortuous in appearance. The basilar artery measured up to 8 mm in diameter in its proximal segment (Figure 1). The tip of the basilar artery reached the suprasellar cistern adjacent to the first segment of the left middle cerebral artery (Figure 2). Such findings were consistent with vertebrobasilar dolichoectasia. The basilar artery was indenting on the brainstem, mainly the pons, but no underlying abnormal signal intensity was noted. There was no secondary obstructive hydrocephalus.

**Figure 3:**
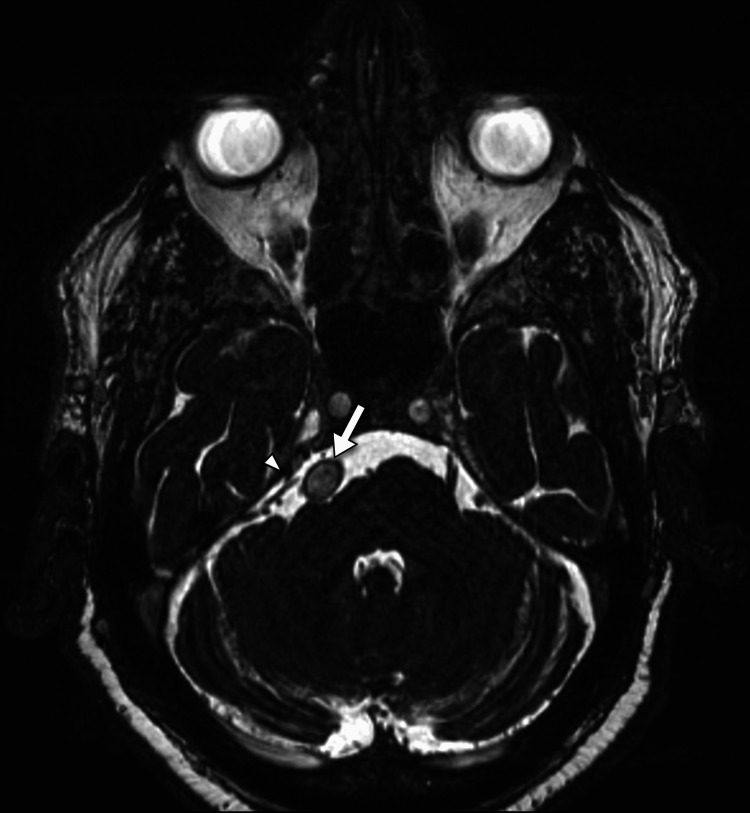
An axial image of the patient's brain MRI in the CISS sequence shows the dilated basilar artery (arrow) compressing and displacing the right trigeminal nerve (arrowhead).

**Figure 4:**
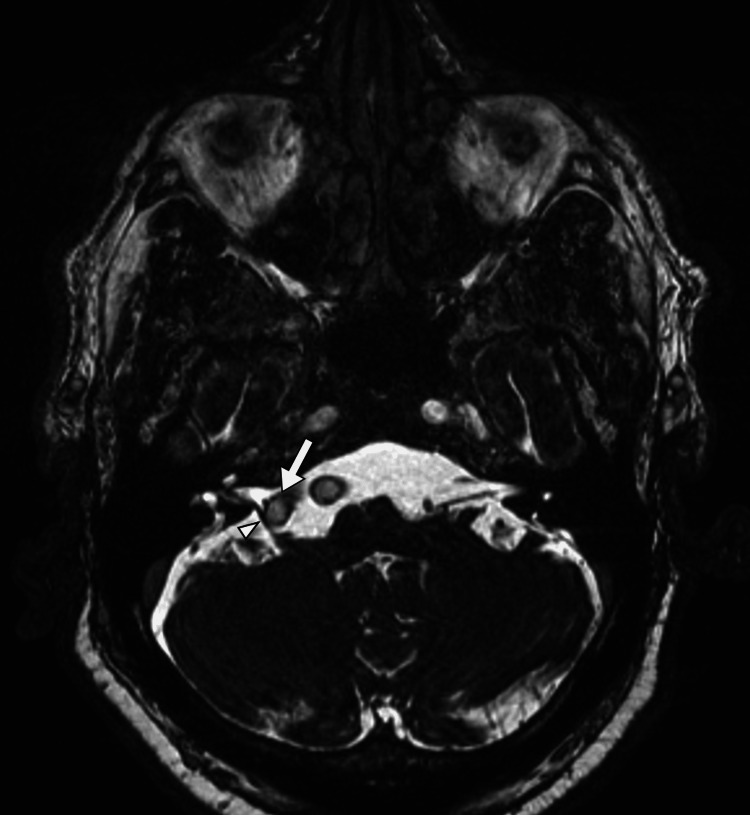
An axial image of the patient's brain MRI in the constructive interference of the CISS sequence shows the dilated right vertebral artery (arrow) compressing and displacing the right facial nerve (arrowhead).

The basilar artery compressed the right trigeminal nerve, which appeared atrophied compared to the contralateral side (Figure 3). The right vertebral artery compressed the right facial nerve in its cisternal segment (Figure 4). There was no vascular occlusion, dissection, or stenosis in the cerebral vasculature.

The clinical diagnosis and the radiological findings were discussed with the patient. Treatment options were provided to the patient, including medical and surgical options. Considering the patient's advanced age and comorbidities, medical treatment was initiated. The patient was given carbamazepine for pain control and was discharged with regular follow-ups in the outpatient clinic. After six months of follow-up, the patient was satisfied with the medical treatment and revealed significant improvement in his symptoms.

## Discussion

We report a case of vertebrobasilar dolichoectasia, an extremely rare cause of trigeminal neuralgia. Dolichoectasia is abnormal elongation and dilation of the arteries, which typically affects the posterior intracranial circulation. While this condition is of unknown etiology, it has been linked to other diseases, including hypertension, collagen vascular disease, polycystic kidney disease, and sickle cell disease. In the present case, the patient had a long-standing history of hypertension and diabetes mellitus. It is postulated that these underlying conditions cause vascular remodeling resulting from an imbalance between antiprotease and metalloproteinase activity [4].

Vertebrobasilar dolichoectasia can be asymptomatic. The clinical manifestations of symptomatic cases vary widely [4]. The most common clinical manifestation is ischemic stroke. Other manifestations include symptoms related to cranial nerve compressions, brainstem compression, hemorrhage, and obstructive hydrocephalus [5]. In the present case, the patient developed trigeminal neuralgia and facial spasms related to neurovascular compression of the trigeminal and facial nerves, respectively. The patient also had compression on the brainstem. However, there was no evidence of an associated brainstem injury or associated hydrocephalus.

In the current case, the patient had a satisfactory response to conservative treatment with carbamazepine for trigeminal neuralgia, and surgical treatment was not performed given the high anesthesia risk for the patient. Vertebrobasilar dolichoectasia is often treated with conservative management [5]. If needed, surgical treatment procedures include ventriculoperitoneal shunt placement for associated hydrocephalus or microsurgery for trigeminal or facial nerve decompression [4,5]. Gamma knife radiosurgery is another treatment approach with favorable outcomes [6].

## Conclusions

Vertebrobasilar dolichoectasia is a rare cause of neurovascular compression on the trigeminal and facial nerves that can lead to trigeminal neuralgia and facial hemispasm. The diagnosis can be readily made by magnetic resonance imaging. Medical management should be attempted first, as it can be effective and obviate the need for surgical treatment, particularly in those patients with comorbidities who are not candidates for surgical interventions.
